# Influence of Dominance and Drift on Lethal Mutations in Human Populations

**DOI:** 10.3389/fgene.2020.00267

**Published:** 2020-04-02

**Authors:** David Waxman, Andrew D. J. Overall

**Affiliations:** ^1^Centre for Computational Systems Biology, ISTBI, Fudan University, Shanghai, China; ^2^School of Pharmacy & Biomolecular Sciences, University of Brighton, Brighton, United Kingdom

**Keywords:** lethal genetic disease, Mendelian disorder, mutation selection drift balance, diffusion analysis, Wright-Fisher model, stochastic population dynamics

## Abstract

We consider disease-causing mutations that are lethal when homozygous. Lethality involves the very strongest form of negative selection, with the selection coefficient against the disease-carrying homozygote having its maximum value of unity. We determine results for the behavior of the frequency of a lethal allele in an effectively infinite population. This includes an estimate of the time it takes to achieve equilibrium, and a description of transient behavior associated with a sudden change in the fitness of the heterozygote. We determine analogous results for a finite population, showing that a lethal disease-causing allele needs to be described by a modified Wright-Fisher model, which deviates from the standard model, where selection coefficients are assumed small compared with 1. We show that a by-product of the dynamics, resulting from the absence of the disease-allele carrying homozygote in adults, is the general constraint that the frequency of the disease-causing allele cannot exceed $\frac{1}{2}$. The results presented in this work should prove useful to a number of areas including analysis of lethal/near lethal mutations in Mendelian disorders and, in particular, for exploring how mutation-selection-drift balance explains the current spectrum of mutation frequencies in humans. While the number of empirical examples of overdominance in lethal genetic disorders is not large, relatively high observed heterozygote frequencies may be a hint of transient heterozygous advantage in nature. For lethal disorders with anomalous frequencies, such as cystic fibrosis and Tay-Sachs, our analysis lends further support to the role that transitory episodes of weak overdominance may play in the evolution of lethal mutations.

## 1. Introduction

The population genetics of single-gene diseases, where a gene is typically considered to have two alleles and three genotypes, is generally an oversimplification. If we consider the textbook example of the ΔF508 mutation of the CFTR gene, which is responsible for the majority of cystic fibrosis cases, we have a gene with a recessive *cc* genotype whose fitness is close to zero, relative to the *CC* and *Cc* genotypes. However, with the discovery of modifier genes that modulate CFTR (Guggino and Stanton, 2006), this gene can no longer be considered within a simple two-allele/three-genotype framework. As of October 2019, the Online Mendelian Inheritance in Man (OMIM) database, reported 6, 516 single-gene phenotypes with known molecular basis. The proportion of these that can be described using a simple two-allele/three-genotype model is likely to be small. Nevertheless, a subset of these Mendelian disorders, which are single-gene diseases, and are caused by an effectively lethal mutation, allows a general analysis based on a two-allele/three-genotype model. Such genes are associated with the very simplest Mendelian diseases, where the disease-causing genotype has a relative fitness that is very small—of the order of 1% or smaller, and hence has a rather accurately known selection coefficient, namely one that is very close to unity, thus representing one of the very strongest forms of negative selection.

The extreme clarity about selection coefficients lent by lethal alleles has been exploited in a recent study by Amorim et al. (2017). These authors explored the frequency distribution of 417 mutations (found within 32 genes) that are known to be recessive lethals. They concluded that most of the mutations do not conform to what is expected from the balance between mutation, purifying selection and random genetic drift. In particular, the authors found that the majority of the mutations observed were at frequencies that were much elevated over what was theoretically expected. The study found some agreement with a specific class of mutations (CpG transitions) but concluded that, on the whole, it was likely that current data reveal an ascertainment bias, where the disease alleles were the ones identified simply by being more frequent by chance. The authors considered the possibility that overdominance may play some part, however, this was not explored theoretically. In light of this work, we present a theoretical investigation of the sensitivity of the mutation-selection dynamic to small changes in fitness of the carrier genotype, in particular when slightly overdominant.

It has been speculated that the unusually high incidence of some lethal disorders, such as cystic fibrosis and Tay-Sachs may have evolved in response to episodes of heterozygous advantage during periods of disease (Yokoyama, 1979; Gabriel et al., 1994). It should be noted, however, that there is currently scant evidence that does not exclude alternative explanations to lethal mutations (Gemmell and Slate, 2006).

When modeling the evolution of allele frequencies, even in large populations like that of *Homo sapiens*, a consideration of the effects of drift may be very significant. However, standard population genetics theory generally incorporates random genetic drift via a Wright-Fisher model (and its diffusion approximation) that is derived under the assumption of *weak selection* (i.e., selection coefficients that are very small compared with 1). The results that follow from this cannot generally be applied in the setting of strong negative selection, namely lethality, by simply setting selection coefficients in weak-selection models to unity. Indeed, a formal analysis of the effects of mutation and random genetic drift in the strong selection context of lethal mutations appears to be lacking in the literature. Here, we present such an analysis. The results should prove useful to a number of areas of interest including analysis of lethal and near lethal mutations in Mendelian disorders, and in exploring how mutation-selection-drift balance explains the current spectrum of mutation frequencies.

We note that there are few empirical examples of overdominance in lethal genetic disorders. This may be a hint of transient heterozygous advantage in nature. For lethal disorders with anomalous frequencies, such as cystic fibrosis and Tay-Sachs, our analysis lends further support to the role that transitory episodes of weak overdominance may play in the evolution of lethal mutations.

## 2. Methods

### 2.1. Description of the Population

**Figure 1:**
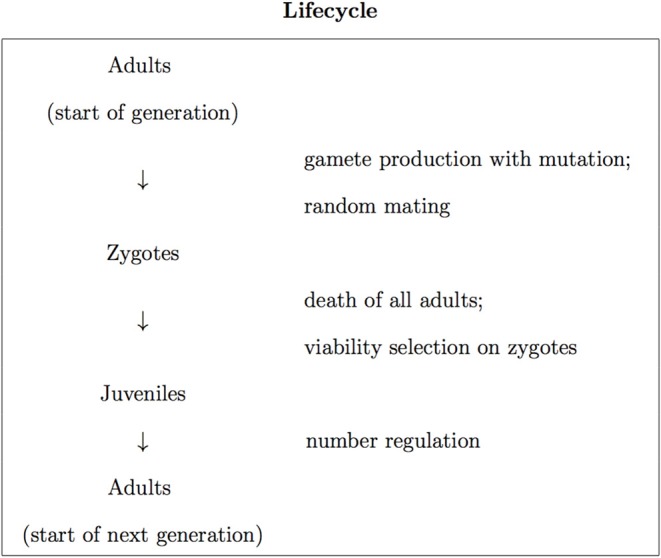
The discrete-generation lifecycle employed in this work.

We base our analysis on a model of a diploid dioecious population with equal sex ratio. This has a discrete-generation lifecycle with census made in adults (Figure 1). We assume that each adult has an equal chance of contributing zygotes to the population, independent of genotype, and that viability selection acts in zygotes at a single biallelic locus. With *A* and *a* denoting the two alleles at the locus, viability selection generally involves the *aa*, *aA* and *aa* genotypes at the locus having different viabilities (i.e., different probabilities to survive to reproductive age), which we write as *V*_*aa*_, *V*_*aA*_, and *V*_*AA*_, respectively. Using *AA* as the reference genotype, we define the relative fitnesses of the three genotypes as $w_{aa}=\frac{V_{aa}}{V_{AA}}$, $w_{aA}=\frac{V_{aA}}{V_{AA}}$, and $w_{AA}=\frac{V_{AA}}{V_{AA}}$. We follow a common way of parameterizing the relative fitnesses, in terms of a selection coefficient, *s*, which determines the difference in relative fitness between the two homozygotes, and a dominance coefficient, *h*, which determines the relative fitness of the heterozygote. This involves writing the relative fitnesses in terms of *s* and *h* as *w*_*aa*_ = 1 − *s*, *w*_*aA*_ = 1 − *hs*, and *w*_*AA*_ = 1. In the present work we take the *a* allele to be disease-causing in the sense that it is lethal in homozygous form, which entails *s* = 1. This leads to the relative fitnesses of the three genotypes being given by

(1) $$\begin{array}{l} w_{aa}=0,\;w_{aA}=1-h,\;w_{AA}=1. \end{array}$$

We note that relative fitnesses are always non-negative, and in the present work, where there is a lethal genotype, this leads to the dominance coefficient, *h* having restricted values so that *w*_*aA*_ = 1 − *h* ≥ 0. The possible values of *h* are thus given by

(2) $$\begin{array}{l} -\infty <h\leq 1. \end{array}$$

The allele of interest in the present work is the disease-causing *a* allele, and we can classify this in terms of the dominance coefficient, *h*. The *a* allele is completely recessive if *h* = 0, partially recessive if 0 < *h* < 1 (which includes the case of additivity if $h=\frac{1}{2}$), completely dominant if *h* = 1, and overdominant if *h* < 0. There is no possibility of underdominance (*h* > 1) since the fitness of the heterozygote cannot lie below that of the lethal (*aa*) genotype.

We incorporate mutation into the model, taking it to be one-way, from the wild type allele to the disease-causing allele. In any generation, each *A* allele in the population has probability *u* of undergoing mutation to the *a* allele:

(3) $$\begin{array}{l} A\underset{u}{\to }a \end{array}$$

and each *A* allele remains unchanged with probability 1 − *u*.

We shall usually write the frequency of the disease-causing *a* allele in a particular generation, termed the *present* generation, as *X*, and use *X*′ to denote its value in the following generation. However, when time is important we shall use *X*_*t*_ to denote the frequency of the *a* allele in generation *t*.

We incorporate number regulation into the lifecycle, where the juveniles in the population are non-selectively thinned, with their number reduced to the number of adults at the start of the generation. The individuals that are present in the population after the thinning process become the adults of the next generation (Figure 1).

In what follows we shall consider stationary and transient behaviors, in the context of effectively infinite and finite populations. We shall investigate how different values/behaviors of the dominance coefficient, *h*, influence various results.

### 2.2. Model for an Effectively Infinite Population

In a very large population, random genetic drift (due to number regulation) plays a negligible role in the lifecycle. The frequency of the disease-causing allele can then be treated as behaving deterministically, and as far as the frequency is concerned, the number of adults in the population is effectively infinite. In what follows, we shall use the shorthand *infinite population* to describe a population of effectively infinite size. We show in Part A of the Supplementary Material that for an infinite population, the rule that relates the frequency in the next generation (*X*′) to that of the present generation (*X*) is given by

(4) $$\begin{array}{l} X^{\prime }=X+F\left(X\right) \end{array}$$

where

(5) $$\begin{array}{l} F\left(x\right)=\frac{\left(1-h\right)u-\left[h-\left(2-3h\right)u\right]x-\left(1-2h\right)\left(1-u\right)x^{2}}{\left[1+\left(1-2h\right)u\right]+\left(1-2h\right)\left(1-u\right)x}. \end{array}$$

The function *F*(*x*) has the interpretation as the deterministic evolutionary force that acts on the frequency of the disease-causing allele (the *a* allele) in a very large population, when the frequency has the value *x*. If, in a given generation, this force is non-zero then the frequency will be different in the following generation.

We give the full form of *F*(*x*) in Equation (5), and later the full form of the corresponding equilibrium frequency (in Equation 9), since these are quite sensitive to the precise values of the parameters *h* and *u*.

A particular example of Equations (4) and (5) is for a recessive lethal allele, when mutation is neglected. This has *h* = 0 and *u* = 0, in which case the evolutionary force in Equation (5) becomes $F\left(x\right)=-\frac{x^{2}}{1+x}$. From Equation (4) we then obtain $X^{\prime }=\frac{X}{1+X}$ which is a textbook example of the evolution of a lethal allele (Hedrick, 1984).

### 2.3. Wright-Fisher Model for a Finite Population

An infinite population is governed by a deterministic equation of the general form of Equation (4), namely *X*′ = *X* + *F*(*X*). When selection is weak, corresponding to |*F*(*x*)| ≪ 1 for all *x*, the behavior of the frequency of the *a* allele in a finite population, under a Wright-Fisher model (Fisher, 1930; Wright, 1931), is governed by the stochastic equation

(6) $$\begin{array}{l} X^{\prime }=\frac{\mathrm{Bin}\left(2N,X+F\left(X\right)\right)}{2N}\;\begin{array}{c} \text{Wright-Fisher model}\\ \text{for weak selection} \end{array} \end{array}$$

where, in the notation adopted in this work, Bin(*n, p*) denotes a binomial random *number* (*not* a distribution), and gives the random number of successes on *n* independent trials, each of which has probability *p* of success.

However, when considering the evolution of lethal mutations we need to modify the above Wright-Fisher model so it incorporates strong selection. This results in a modified Wright-Fisher model. The model we shall present is designed to be appropriate for a modern, post-industrial human population, where fertility is approximately two offspring per couple (Hamilton et al., 2012) as occurs, for example, in the USA. Details of the model are given in Part B of the Supplementary Material.

The conventional Wright-Fisher model is based on the strong assumption that all randomness arises solely in the non-selective thinning of the population to the census population size^1^. This means, in particular, that selection is treated as a deterministic process, amounting to the population being effectively infinite during the time that selection occurs within the lifecycle (the zygotic stage). For humans in modern post-industrial populations, the number of offspring produced is typically little more than that required to replace the population (Hamilton et al., 2012). Thus, the number of zygotes produced is similar in number to the number of adults (i.e., similar to the census size). To transparently avoid any possible consequences of an effectively infinite number of zygotes, we have used an explicitly probabilistic treatment of selection, where we have strongly limited the number of zygotes produced. However, as we show in Part B of the Supplementary Material, for most practical purposes there is a negligible difference between such a model, and the model where selection is treated as acting deterministically.

The main difference that arises because selection is not weak, but strong (because of lethality of one genotype), is that selection cannot be directly approximated as acting at the level of alleles (which is possible when selection is weak). Implementing viability selection at the actual level at which it acts, namely genotypes, we find that although the equation *X*′ = *X* + *F*(*X*) applies to the frequency of the disease-causing allele in an infinite population (see Equations 4 and 5), we cannot simply extend this equation to become an equation describing a finite population, by using the weak-selection result (Equation 6). Rather, we find (see Part B of the Supplementary Material for details) that the frequency of the lethal genotype obeys the stochastic equation

(7) $$\begin{array}{l} X^{\prime }=\frac{\mathrm{Bin}\left(N,2X+2F\left(X\right)\right)}{2N}\;\begin{array}{c} \text{Wright-Fisher model}\\ \text{for a lethal genotype} \end{array} \end{array}$$

where *F*(*x*) is given in Equation (5). We shall often refer to Equation (7) as the Wright-Fisher model describing a lethal genotype.

A comparison of the standard weak-selection Wright-Fisher model (Equation 6), and the Wright-Fisher model describing a lethal genotype (Equation 7), indicates differences in the placement of factors of 2 in *both* arguments of the binomial random numbers present [the Bin(*n, p*)]. Despite these differences, it may be verified that when *N* → ∞, in which case $\frac{\mathrm{Bin}\left(N,p\right)}{N}\to p$, the factors of 2 cancel and Equation (7) reduces to *X*′ = *X* + *F*(*X*). Thus, the quantity *F*(*X*) appearing in Equation (7) continues to have the interpretation as the deterministic evolutionary force acting in an infinite population.

Simulations contained in the Supplementary Material were performed using MATLAB with the Statistics Toolbox. The code is available at https://github.com/AndyOverall/Overdominance.

#### 2.3.1. General Implication of Lethality

We wish to point out one general implication of lethality within the context of a biallelic locus. This is that lethality of one homozygote *generally* constrains the frequency of the lethal allele in adults, such that independent of any model used, and independent of the size of the population, the frequency of the lethal allele can never exceed $\frac{1}{2}$. To see this we use a simple gene counting argument, as follows. We note that the lethal *a* allele only appears in heterozygote adults, but the wild type (*A*) allele appears in both viable homozygote adults and heterozygote adults. Hence, the frequency *X* of the *a* allele in adults is $X=\frac{\text{number of }aA\text{ adults}}{2\times \text{ number of }aA\text{ adults}+2\times \text{ number of }AA\text{ adults}}$. The right hand side of this equation can be written as $1/\left(2+\frac{\text{number of }AA\text{ adults}}{\text{number of }aA\text{ adults}}\right)$ which is *always* less than or equal to one half, thus generally

(8) $$\begin{array}{l} X\leq \frac{1}{2}. \end{array}$$

A particular implication of Equation (8) is that irrespective of the fitness of the heterozygote, it is impossible for there to be a selective sweep of the lethal *a* allele to fixation, and at most the frequency of this allele can only reach $\frac{1}{2}$.

We note that the Wright-Fisher model for a lethal genotype (Equation 7), involves a binomial random number of the form Bin(*N, p*), corresponding to the random number of successes on *N* independent trials. Since the maximum possible number of such successes is *N* we have Bin(*N, p*) ≤ *N*. This has the consequence for Equation (7) that $X^{\prime }\leq \frac{N}{2N}$, i.e., $X^{\prime }\leq \frac{1}{2}$. The *N* independence of this result indicates that independent of the population size (finite or infinite) the constraint of Equation (8), on the frequency of the lethal allele in adults, will apply. It is necessary (and reassuring) to see this constraint directly manifested in the Wright-Fisher model that was constructed for the problem at hand. Generally, any valid model describing a lethal allele must exhibit such a constraint on the frequency of the lethal allele.

## 3. Results

### 3.1. Results for an Infinite Population

#### 3.1.1. Equilibrium Frequency

Equation (4) describes an infinite population. A property of this equation, with *F*(*x*) given by Equation (5), is that the frequency of the *a* allele approaches a stable equilibrium value, which we shall denote by $\hat{X}$. In Part A of the Supplementary Material we give exact and approximate results for the equilibrium frequency following from Equation (4). In particular the equilibrium frequency has the exact form

(9) $$\begin{array}{l} \hat{X}=\frac{2\left(1-h\right)u}{h+\left(2-3h\right)u+\sqrt{h^{2}{\left(1+u\right)}^{2}+4\left(1-2h\right)u}}. \end{array}$$

This result for the equilibrium frequency, $\hat{X}$, applies for the full range of parameters −∞ < *h* ≤ 1 and 0 ≤ *u* ≤ 1 and is consistent with the results given in textbooks. In the case where *h* is small and positive, in the range $\sqrt{u}\ll h\ll 1$, we have the approximation

(10) $$\begin{array}{l} \hat{X}≃\frac{\left(1-h\right)u}{h}. \end{array}$$

In the case of a fully recessive *a* allele (i.e., *h* = 0), the equilibrium frequency of the *a* allele is

(11) $$\begin{array}{l} \hat{X}=\frac{\sqrt{u}}{1+\sqrt{u}}. \end{array}$$

Lastly, in the case of overdominance, where *h* is negative (*h* = −|*h*|), and has a small magnitude in the range $\sqrt{u}\ll |h|\ll 1$, the equilibrium frequency of the *a* allele has an approximate value that is independent of *u* and given by

(12) $$\begin{array}{l} \hat{X}≃\frac{\left(-h\right)}{1-2h}\equiv \frac{\left|h\right|}{1+2|h|}. \end{array}$$

This last result can be determined from Equation (3.4) of the textbook by Gillespie (2004) which gives, in the notation of the present work, the equilibrium frequency of the *A* allele.

The above results indicate that for some degree of recessiveness (i.e., 0 ≤ *h* < 1) the equilibrium attained is primarily the result of a balance between mutation and heterozygote selection, while for overdominance (*h* < 0) it follows that $\hat{X}$ has a very weak dependence on mutation, and is largely determined by the elevated fitness of heterozygotes over homozygotes. An extreme example of the above result is in the case of a negligibly small mutation rate, where $\hat{X}$ takes the very simple form

(13) $$\begin{array}{l} \underset{u\to 0}{\lim}\hat{X}=\left\{ \begin{array}{cc} 0, & \text{for }h\geq 0,\\ \frac{\left|h\right|}{1+2|h|}, & \text{for }h<0. \end{array}\right. \end{array}$$

This result can be simply derived from the *u* = 0 limit of Equations (4) and (5).

**Figure 2:**
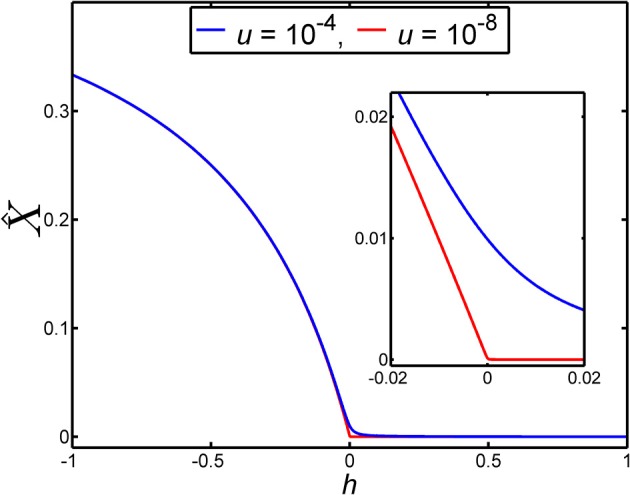
The equilibrium frequency of a lethal allele in a very large population, $\hat{X}$, depends on the dominance coefficient, *h*, according to Equation (9). In this figure the dependence of $\hat{X}$ on *h* is illustrated for two different values of the mutation rate, *u*. The blue curve gives the dependence of $\hat{X}$ on *h* when the mutation rate is *u* = 10^−4^. The red curve gives the dependence of $\hat{X}$ on *h* when the mutation rate is *u* = 10^−8^. The inset is an enlargement of main panel over the range −0.02 ≤ *h* ≤ 0.02, to show the detailed behavior of $\hat{X}$ over this small range of *h*. We note that the larger mutation rate (*u* = 10^−4^) was chosen for the purposes of visualization rather than realism.

When the dominance coefficient, *h*, lies in the vicinity of *h* = 0 the results for the equilibrium frequency, $\hat{X}$, given in Equations (10)–(12) exhibit strongly differing behaviors (see Figure 2).

From Figure 2 it can be seen that equilibrium frequency, $\hat{X}$, is not particularly sensitive to the value of the dominance coefficient, *h*, when it lies in the range 0 ≤ *h* ≤ 1. This arises for this range of *h* because $\hat{X}$ is strongly limited by the value of the mutation rate, *u*. Indeed, for values of *h* that are large compared with $\sqrt{u}$ we have $\hat{X}$ approximately proportional to *u* (see Equation 10) while for values of *h* that are small compared with $\sqrt{u}$ we have $\hat{X}$ approximately proportional to $\sqrt{u}$ (see Equation 11). However, in the region where the disease-causing allele exhibits overdominance (*h* < 0), and the degree of overdominance, |*h*|, is large compared with $\sqrt{u}$, we find $\hat{X}$ is approximately independent of *u* (see Equation 12). This is a feature that is apparent in Figure 2. Given a mutation rate of e.g., *u* = 10^−5^ or smaller, it follows that almost any level of overdominance of the disease-causing allele leads to an $\hat{X}$ in a large population that is anomalously large relative to the mutation-limited values of $\hat{X}$ that apply when this allele is recessive.

The way the equilibrium frequency, $\hat{X}$, depends on the dominance coefficient, *h*, means that if *h* is changed over a numerically small range in the vicinity of *h* = 0, but the range of *h* is large compared with the mutation rate, *u*, then $\hat{X}$ can vary substantially. For example, for *u* = 10^−5^, changing the dominance coefficient from *h* = 0.01 to *h* = −0.01, thereby causing the heterozygote to change from being recessive to being overdominant, causes $\hat{X}$ to change from a value that we can write as $\hat{X}≃91\times u$ to the value $\hat{X}≃1071\times u$ (see Figure 2). That is, an ~2 percent increase in the relative fitness of the heterozygote, 1 − *h*, causes a large increase of roughly one thousand percent in $\hat{X}$. With smaller mutation rates, the percentage increase in $\hat{X}$ will be even larger.

#### 3.1.2. Transient Behavior

##### 3.1.2.1. Scenario

Let us now consider a very large (effectively infinite) population with mutation rate *u*.

We shall consider the following scenario.

1. For a *considerable* time prior to time *t* = 0 the dominance coefficient has the constant value *h* = 0 that corresponds to a completely recessive disease-causing allele.
2. At time *t* = 0, an environmental change discontinuously elevates the value of the heterozygote fitness above 1, driving the disease-causing allele to become overdominant. We write this elevated fitness as 1 − h* with h* negative (h* = −|h*|). This elevated fitness value persists until generation *t*_*f*_.
3. From generation *t*_*f*_ + 1 onwards, the environment reverts back to its original state, with dominance coefficient *h* = 0.

We proceed under the assumption that by the time *t* = 0 is reached the population has come to an equilibrium with a frequency $\hat{X}$ that is appropriate to a dominance coefficient of *h* = 0. Thus, the frequency of the disease-causing allele at time *t* = 0 is given by the infinite population result of Equation (9) for for *h* = 0, i.e., Equation (11), namely $\hat{X}=\frac{\sqrt{u}}{1+\sqrt{u}}≃\sqrt{u}$.

We shall also make the assumption that h* (which is negative) is small, but not too small, in the sense

(14) $$\begin{array}{l} \sqrt{u}\ll |h^{*}|\ll 1. \end{array}$$

This assumption allows us to use some of the approximate results we have presented above, and thereby gain some analytical insights.

Note that the elevated value of the heterozygote fitness after time *t* = 0 causes the frequency to increase after this time. If the time *t*_*f*_ is sufficiently long, then the frequency achieves an equilibrium value (appropriate to dominance coefficient h*) well before time *t*_*f*_, and given by Equation (12), namely ${\hat{X}}^{*}≃\frac{\left|h^{*}\right|}{1+2|h^{*}|}≃|h^{*}|$. Because of Equation (14) it follows that ${\hat{X}}^{*}$ is much greater than the frequency at time *t* = 0, namely $\hat{X}≃\sqrt{u}$. Thus, ${\hat{X}}^{*}$ represents a significant frequency increase over $\hat{X}$.

**Figure 3:**
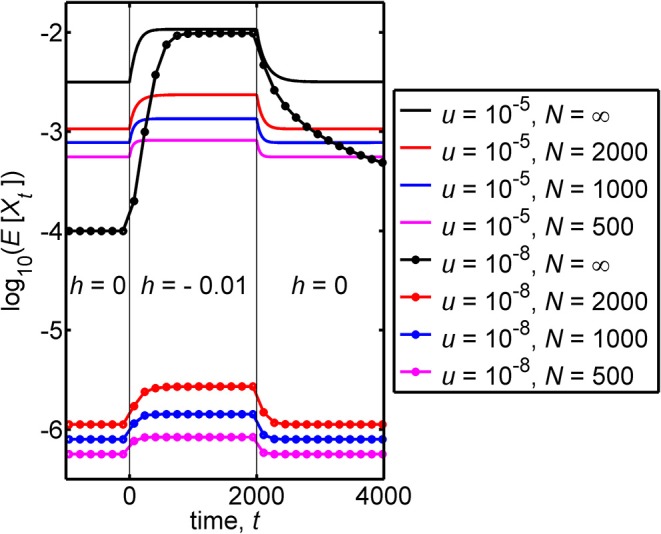
This figure contains plots of the logarithm of the mean frequency of the disease-causing allele, log_10_(*E*[*X*_*t*_]), against the time, *t*. For a large (effectively infinite) population, there are negligible deviations of the frequency from its expected value and *E*[*X*_*t*_] then coincides with the frequency itself, *X*_*t*_. The infinite population results are given by the black curves. Finite population results are given by colored curves. The figure illustrates transient behavior that the frequency can exhibit in populations with different mutation rates, *u*, and different population sizes, *N* when the following are assumed. (i) For a very long time prior to *t* = 0 the relative fitness of the heterozygote has the value 1 (corresponding to a dominance coefficient of 0). (ii) At time *t* = 0 the relative fitness of the heterozygote discontinuously jumps to the value 1.01 (corresponding to a discontinuous jump in the dominance coefficient from 0 to −0.01). (iii) At time *t* = 2, 000, the relative fitness of the heterozygote discontinuously jumps back to the value 1 (corresponding, again to a dominance coefficient of 0). The figure was obtained using Equation (4), for an effectively infinite population, and from the Wright-Fisher model describing a lethal genotype, based on Equation (7). We note that where the curves become flat closely corresponds to the attainment of equilibrium, and values of equilibrium frequencies can be found in Table 1. Additionally, the time to approach equilibrium depends on the pre- and post-jump values of *h*, and each curve takes different times to approach equilibrium. A measure of such times to equilibrium is given in Table 2. The black dotted curve, which applies for *u* = 10^−8^ and *N* = ∞, is the only curve that does not come close to equilibrium at long times. The equilibrium value, that this curve eventually attains, coincides with the value that the curve takes prior to *t* = 0.

Figure 3 illustrates the case where the allele frequency has evolved for a very large number of generations *prior* to time *t* = 0. Substantially before the time *t* = 0 is reached the frequency has achieved the equilibrium value $\hat{X}$ that is appropriate to a dominance coefficient of *h* = 0. For the figure, we chose *t*_*f*_ = 2, 000, and from *t* = 1 to *t* = 2, 000 the dominance coefficient has the value h* = −0.01. Figure 3 illustrates that a relatively small discontinuous change in the heterozygote fitness (in the figure from 1 to 1.01) can, in a large population, lead to a significant increase in the frequency that is subsequently achieved by the disease-causing allele, as shown by the black curves in the figure.

##### 3.1.2.2. Increase of the frequency to equilbrium

For *intermediate times* (1 ≤ *t* ≤ 2, 000) the frequency can be seen in the black curves of Figure 3 to increase and reach an equilibrium, for both mutation rates that were used.

For *u* = 10^−8^, the frequency at time *t* = 0 is $\hat{X}≃1.0\times 10^{-4}$, and this leads, at an intermediate time, to the equilibrium value ${\hat{X}}^{*}≃9.8\times 10^{-3}$ which is ~100 times larger than $\hat{X}$.

To make comparisons, it is useful to have a measure of the *time* to equilibrium. It is, however, hard to provide a precise definition of the time to equilibrium. We thus introduce the well-defined time it takes the frequency to go *half* the distance from its initial value to its final equilibrium value, and we denote this time by *T*_1/2_. Thus, for intermediate times, *T*_1/2_ is the time taken for the frequency to go from its initial value (at time *t* = 0), namely $\hat{X}$, to its ‘mid-point value' $\hat{X}+\left({\hat{X}}^{*}-\hat{X}\right)/2=\left(\hat{X}+{\hat{X}}^{*}\right)/2$. We call *T*_1/2_ the “half time to equilibrium,” and this can be calculated from the analytical solution of the frequency (see Part A of the Supplementary Material) or estimated from Figure 3. For intermediate times the half time to equilibrium can be approximated by

(15) $$\begin{array}{l} T_{1/2}≃\frac{\text{ln }\left(\frac{\left|h^{*}\right|}{1+2|h^{*}|}\frac{1}{\sqrt{u}}\right)}{\text{ln }\left(1+|h^{*}|\right)} \end{array}$$

(see Part A of the Supplementary Material for details). The time to achieve equilibrium, if required, can be very roughly estimated as a multiple of *T*_1/2_, for example 3 × *T*_1/2_, but if this time is important then justification is required for the multiple of *T*_1/2_ used.

For h* = −0.01 and *u* = 10^−8^, Equation (15) closely agrees with the exact result *T*_1/2_ = 461 generations, which is consistent with the relevant infinite population (black) curve in Figure 3.

For *u* = 10^−5^ the frequency at time *t* = 0 is $\hat{X}≃3.2\times 10^{-3}$, the equilibrium value achieved at an intermediate time is ${\hat{X}}^{*}=1.1\times 10^{-2}$ and the “half time” to equilibrium is *T*_1/2_ = 114 generations, which is again close to the approximation in Equation (15).

##### 3.1.2.3. Decrease of the frequency to equilbrium

For *later times* (*t* > 2, 000) the frequency can be seen in Figure 3 to decrease. For a mutation rate of *u* = 10^−5^ an equilibrium value of the frequency is achieved, but for *u* = 10^−8^ no equilibrium value is achieved up to time *t* = 4, 000.

For later times the initial value is ${\hat{X}}^{*}$ and the final equilibrium value is $\hat{X}$, which is a simple interchange of the initial and equilibrium value used for intermediate times. However, the behavior of the frequency at later times is not the ‘mirror image' of the behavior at intermediate times. The value of the dominance coefficient, at intermediate times (1 ≤ *t* ≤ 2, 000) during the approach to equilibrium, is −0.01, but by contrast, at later times (*t* > 2, 000) the value of the dominance coefficient during the approach to equilibrium is 0. This difference in dominance coefficients, during the approach to equilibrium, leads to different “half times to equilibrium.” For later times, for parameter-values similar to those adopted, the half time to equilibrium can be well-approximated by

(16) $$\begin{array}{l} T_{1/2}≃\frac{\ln\left(1+\frac{2\sqrt{u}}{\left|h^{*}\right|}-\frac{2u}{{\left(h^{*}\right)}^{2}}\right)}{2\sqrt{u}} \end{array}$$

(see Part A of the Supplementary Material for details) which has a quite different form to that of Equation (15).

For *u* = 10^−8^ we find that the time it takes, from the time *t* = 2, 000, to achieve half equilibrium is *T*_1/2_ = 100 generations, while for *u* = 10^−5^ it is *T*_1/2_ = 59 generations, and these values of *T*_1/2_ are close to the approximation in Equation (16). We note that the two values of *T*_1/2_ for later times are smaller than the corresponding values for intermediate times.

### 3.2. Results for a Finite Population

We can obtain results for the Wright-Fisher model described by Equation (7), which is a discrete time Markov chain where some exact results are known (Haigh, 2013) and for which many results can be numerically calculated. Before we give results based on numerical calculations, we note that analytical insight into the Wright-Fisher model, and the phenomena occurring in a finite population, can be gained using a *diffusion approximation* of this model (Kimura, 1955). The diffusion approximation treats both the allele frequency and time as continuous quantities, and replaces the random frequency *X*_*t*_ of the Wright-Fisher model by a continuous function of continuous time, which we write as *X*(*t*). This approximation results in the replacement of the discrete distribution of the Wright-Fisher model by a probability density of the frequency, which at time *t* and frequency *x* we write as ϕ(*x, t*). The probability density obeys the diffusion equation

(17) $$\begin{array}{l} -\frac{\partial \phi \left(x,t\right)}{\partial t}=-\frac{1}{2}\frac{\partial^{2}}{\partial x^{2}}\left[V\left(x\right)\phi \left(x,t\right)\right]+\frac{\partial }{\partial x}\left[F\left(x\right)\phi \left(x,t\right)\right] \end{array}$$

(see Part C of the Supplementary Material for details) where

(18) $$\begin{array}{l} V\left(x\right)=\frac{\left[x+F\left(x\right)\right]\left[1-\left(2x+2F\left(x\right)\right)\right]}{2N} \end{array}$$

is the “infinitesimal variance” associated with the model, while *F*(*x*) is the deterministic force given in Equation (5).

Some intuition about the phenomena occurring in a finite population with a lethal genotype can be gained from the form of the coefficients *V*(*x*) and *F*(*x*) that appear in the diffusion equation (Equation 17). We proceed, noting that many of the phenomena associated with the frequency of a lethal disease-causing allele occur at low frequencies (*x* ≪ 1), and so look at the forms of *V*(*x*) and *F*(*x*) at small *x*. On neglecting small terms of order *x*^2^, *xu* and *u*^2^ we find that for small *x*

(19) $$\begin{array}{l} V\left(x\right)≃\frac{\left(1-h\right)\left(x+u\right)}{2N}\text{and }F\left(x\right)≃u\left(1-h\right)-hx\text{.} \end{array}$$

We assume that *h* is not equal to 1, so the disease-causing allele is not fully dominant. Then the result in Equation (19) for *V*(*x*) at small *x* tells us that there are fluctuations in the allele frequency that are characterized by $\sqrt{V\left(x\right)}≃\sqrt{\left(1-h\right)\left(x+u\right)/\left(2N\right)}$ which are small, but which persist even at zero frequency. This is unlike the standard (i.e., weak selection) Wright-Fisher model, where the corresponding result is $\sqrt{V\left(x\right)}≃\sqrt{x/\left(2N\right)}$, which ultimately vanishes at zero frequency. For *x*≫*u* the form of *V*(*x*) in Equation (19) behaves as $\sqrt{\left(1-h\right)x/\left(2N\right)}$ suggesting that such a population has fluctuations appropriate to a population of size *N*/(1 − *h*) which is *larger* than *N*, hence resulting in *smaller* fluctuations than those expected for a standard Wright-Fisher model of population size *N*. The result in Equation (19) for the force *F*(*x*) acting on the allele frequency tells us that at zero frequency the force is approximately *u*(1 − *h*). This is positive, and hence has the tendency to push the frequency to positive values, as we would expect mutation to do. However it is reduced by a factor equal to the heterozygote fitness, 1 − *h*. Indeed, since the non-lethal aspect of selection takes place in heterozygotes, it is not surprising to see the relative fitness 1 − *h* of the heterozygotes influencing the fluctuations in the frequency and also manifesting itself in the force acting on the allele frequency.

#### 3.2.1. Stationary Distribution

Under the Wright-Fisher model, the fraction of a very long period of time spent by the population at a particular frequency is given by the value of the stationary distribution at this frequency (Gillespie, 2004). The expected value of the frequency in this distribution is the finite population analog of the equilibrium frequency in an infinite population.

For the Markov chain of Equation (7) we write the stationary distribution as **π**. This is a column vector with elements π_*n*_ (*n* = 0, 1, 2, …, 2*N*) that is the unique solution to

(20) $$\begin{array}{l} \text{W}\pi =\pi \text{ with }\pi_{n}\geq 0\text{ and }\overset{2N}{\underset{n=1}{\sum }}\pi_{n}=1 \end{array}$$

where **W** is the transition matrix of the Markov chain of Equation (7) (see Part D of the Supplementary Material for details of the Wright-Fisher model as a Markov chain). For the problem at hand, the elements of π_*n*_ with *n* > *N* are zero. We can numerically determine the stationary distribution, **π**, and from this distribution numerically determine the value of the mean frequency, which we denote by *E*_stat_[*X*]. In Table 1 we illustrate the dependence of *E*_stat_[*X*] on the parameters *u*, *N* and *h*. The results of Table 1 suggest that *E*_stat_[*X*] is: (i) an increasing function of *N*, (ii) a decreasing function of *h*, (iii) an increasing function of *u*.

**Table 1:** Finite and infinite population frequencies when at stationarity/equilibrium.

**Mutation rate, *u***	**Population size, *N***	**Dominance coefficient, *h***	***N* × *h***	**log_10_ (*E*_stat_[*X*])**	***E*_stat_[*X*]/*u***
10^−8^	500	−0.01	−1	−2.373	4.238 × 10^5^
		−0.001	−0.1	−6.077	8.375 × 10^2^
		0	0	−6.248	5.650 × 10^1^
		0.001	0.1	−6.390	4.071 × 10^1^
		0.01	1	−7.061	8.693
	1,000	−0.01	−10	−1.093	8.071 × 10^6^
		−0.001	−1	−5.846	1.426 × 10^2^
		0	0	−6.098	7.971 × 10^1^
		0.001	1	−6.292	5.106 × 10^1^
		0.01	10	−7.054	8.839
	2,000	−0.01	−50	−1.085	8.220 × 10^6^
		−0.001	−5	−5.567	2.709 × 10^2^
		0	0	−5.949	1.125 × 10^2^
		0.001	5	−6.208	6.193 × 10^1^
		0.01	50	−7.050	8.917
	∞	−0.01		−1.079	8.333 × 10^6^
		−0.001		−2.009	9.805 × 10^5^
		0		−4.000	9.999 × 10^3^
		0.001		−6.004	9.899 × 10^1^
		0.01		−7.046	9.000
10^−5^	500	−0.01	−1	−1.114	7.692 × 10^3^
		−0.001	−0.1	−3.087	8.186 × 10^1^
		0	0	−3.254	5.569 × 10^1^
		0.001	0.1	−3.394	4.0333 × 10^1^
		0.01	1	−4.061	8.687
	1,000	−0.01	−10	−1.091	8.108 × 10^3^
		−0.001	−1	−2.869	1.151 × 10^2^
		0	0	−3.111	7.751 × 10^1^
		0.001	1	−3.299	5.027 × 10^1^
		0.01	10	−4.054	8.832
	2,000	−0.01	−50	−1.085	8.230 × 10^3^
		−0.001	−5	−2.628	2.355 × 10^2^
		0	0	−2.972	l.066 × 10^2^
		0.001	5	−3.219	6.043 × 10^1^
		0.01	50	−4.050	8.910
	∞	−0.01		−1.079	8.343 × 10^3^
		−0.001		−1.970	1.071 × 10^3^
		0		−2.501	3.152 × 10^2^
		0.001		−3.042	9.075 × 10^1^
		0.01		−4.046	8.992

*In this table, we give results for the logarithm of the mean allele frequency in the stationary distribution of a finite population, log_10_(E_stat_[X]), for different values of mutation rate, u, the population size, N, and the dominance coefficient, h. For a large (effectively infinite) population, there are negligible deviations of the frequency from its expected value and E_stat_[X] coincides with the equilibrium frequency $\hat{X}$. To aid comparison, we have given $\hat{X}$ in the table, listed under N = ∞. Of interest is the relationship between E_stat_[X] and u (see Discussion) and we provide a column in the table containing the ratio E_stat_[X]/u*.

#### 3.2.2. Transient Behavior

For a finite population, we have so far considered the stationary distribution of the disease allele's frequency. Let us now try to get some insight into the transient behavior that also occurs in a finite population. We assume the same scenario of changes of the dominance coefficient *h* as before (see section 3.1.2).

We proceed, taking the frequency at time *t* = 0 to be described by the stationary distribution corresponding to a dominance coefficient of 0. The distribution then evolves after time *t* = 0 when subject to a dominance coefficient of h* which is negative. A basic characterization of this problem is in terms of the expected value of the frequency. We note that compared with the infinite population result, we now have an additional parameter in the problem, namely the population size, *N*, and results will depend on the value adopted for this parameter. We investigate the basic trends associated with finite *N* in the regime *Nu* ≪ 1 by considering two different mutation rates and three different values of the population size, *N*. In Figure 3 the logarithm of the mean allele frequency, log_10_(*E*[*X*_*t*_]), is plotted (in colored curves) against the time, *t*.

In Figure 3 the behavior of the finite population results for *E*[*X*_*t*_] can be seen to be qualitatively similar to those of an infinite population, but quantitatively different. Mean frequencies in a finite population are, from the figure, smaller than the corresponding equilibrium frequencies of an infinite population. The corresponding “half-times to equilibrium,” *T*_1/2_, which^2^ can be seen to differ from the corresponding infinite population results. To clarify this aspect we give the values of *T*_1/2_ in Table 2.

**Table 2:** Half times to equilibrium in a finite population.

**Mutation rate, *u***	**Population size, *N***	**Pre-jump dominance coefficient**	**Post-jump dominance coefficient**	***T*_1/2_**
10^−8^	500	0	−0.01	37
	1,000	0	−0.01	60
	2,000	0	−0.01	103
	∞	0	−0.01	461
	500	−0.01	0	27
	1,000	−0.01	0	37
	2,000	−0.01	0	49
	∞	−0.01	0	100
10^−5^	500	0	−0.01	36
	1,000	0	−0.01	56
	2,000	0	−0.01	88
	∞	0	−0.01	114
	500	−0.01	0	27
	1,000	−0.01	0	36
	2,000	−0.01	0	46
	∞	−0.01	0	59

*In this table, we give results for half time to equilibrium, T_1/2_, for different values of mutation rate, u, the population size, N, and the dominance coefficient, h. To aid comparison, we also give T_1/2_ in an effectively infinite population, which is listed under N = ∞*.

For the pattern of environmental changes we have considered, where *h* is initially 0 and discontinuously jumps to −0.01, or the reverse of this, the results of Table 2 suggest that *T*_1/2_ is: (i) an increasing function of *N*, (ii) a decreasing function of *u*, (iii) a jump from *h* = 0 to *h* = −0.01 leads to a larger *T*_1/2_ than a jump from *h* = −0.01 to *h* = 0.

## Discussion

In this work we have provided an analysis of the implications of lethal mutations in both effectively infinite and finite populations.

For an effectively infinite population, we have given the general form for the deterministic evolutionary force which acts in such a system, along with the equilibrium frequency. We have also provided some illustrations and a characterization of the transient behavior of the frequency when the fitness of the heterozygote discontinuously changes. For a finite population, we have provided the appropriate (i.e., a modified) Wright-Fisher model and discussed some features that become apparent under a diffusion approximation. We have presented properties of a finite population, such as the stationary distribution and its transient behavior.

For populations of finite size, Wright-Fisher models (and their diffusion approximations) have often been employed in describing the evolution of a focal allele (see e.g., Ewens, 2004). One assumption that is typically made when taking this approach is that selection is a weak evolutionary force, in the sense that selection coefficients are small compared with 1. However, the assumption of weak selection becomes untenable for lethal mutations; lethality represents the strongest level of selection against one genotype. Thus an important consideration with lethality, is the explicit need to treat the action of selection on genotypes, rather than on alleles. This would appear to make the analysis of lethal mutations significantly more complicated than when selection acts weakly on all genotypes (in which case a description in terms of single allele frequency suffices). However, perhaps surprisingly, lethal selection has no more complexity than weak selection. This arises for a single locus with two alleles since a description of the population is generally required in terms of three genotype frequencies, but the three genotype frequencies add to unity so just two are independent, and when there is also lethality of one homozygote, this allows elimination of one of the two independent genotype frequencies, with the substantial simplification that just a single frequency is required to describe the population. Thus, lethality of one genotype has the effect of simplifying the model. The multinomial distribution that is required to relate genotype frequencies in adjacent generations under more general schemes of selection (Nagylaki, 1992) collapses to a binomial distribution, thereby making the problem mathematically no more complex than a weak selection problem, which is also described by just a single frequency and also involves a binomial distribution.

The absence of the homozygous disease genotype in adults has the general consequence that the frequency of the mutant allele is, under all circumstances, constrained to have a frequency in the adult population that is $⩽\frac{1}{2}$. Thus, while it might be viewed as *improbable*, but not impossible, that a lethal mutation can rise to a frequency above $\frac{1}{2}$, the analysis presented in this work indicates that this can never be the case. If a supposedly lethal allele is seen at a frequency in excess of $\frac{1}{2}$, then it can be concluded that the allele is not lethal (or perhaps that a two allele/three genotype model is an oversimplification of the real situation).

In Table 1 we gave expected values of the frequency of the disease-causing allele, in a finite population, in the stationary distribution, *E*_stat_[*X*]. These can be seen to be approximately proportional to the mutation rate, *u*, when *h* > 0. For example, when *N* = 10^3^ and *h* = 0.01, with *u* = 10^−8^ we have *E*_stat_[*X*]/*u* ≃ 8.839, while for the same *N* and *h*, but *u* = 10^−5^ we obtain almost the same ratio *E*_stat_[*X*]/*u* ≃ 8.832. Table 1 applies when *Nu* ≪ 1 and the nature of lethal mutations, to rarely make an appearance within such a finite population, accounts for the observed proportionality when *h* > 0. However, the ratio becomes very sensitive to *N* when *h* < 0, corresponding to overdominance of the disease-causing allele. For example, Table 1 shows that when *u* = 10^−8^ and *h* = −0.01, that with *N* = 500 we have $E_{\mathrm{stat}}\left[X\right]/u≃4.238\times 10^{5}$, however for the same *u* and *h* but *N* = 10^3^ we obtain a ratio that is almost 20 time larger: $E_{\mathrm{stat}}\left[X\right]/u≃8.071\times 10^{6}$. We infer that with weak overdominance, the lethal allele can, in larger populations, reach higher frequencies that are more in line with some lethal disorders.

In a recent study on lethal mutations by Amorim et al. (2017), these authors found that of the four mutation types responsible for lethality they studied, the lower the mutation rate, the greater the observed frequency differed from their expectations, based upon mutation-selection-drift balance. In particular, for three of the four mutation types, the observed frequency was significantly higher than the theoretical expectation. Here we have found, for a large (effectively infinite) population size, that as the mutation rate decreases, the sensitivity of the equilibrium allele frequency to overdominance increases (Figure 2). Importantly, this relationship between equilibrium frequency and mutation rate is found within a very small window around *h* = 0. When *h* > 0, the equilibrium frequency is proportional to *u* (see $\hat{X}$, Table 1), whereas when *h* < 0, the equilibrium frequencies of lethal alleles are (to leading order) independent of the mutation rate, being a simple algebraic function of *h* (see Equation 13). Consequently, for lethal alleles with a low mutation rate, even very weak overdominance can result in highly inflated equilibrium frequencies, that largely escape mutation limitation. It may be of some relevance that when considering the transient behavior of the mutation frequency (Figure 3), the time for the mutation to approach equilibrium subsequent to a period of overdominance can be considerable: e.g., of the order of 500 generations for some of the parameter values considered here. Realistically, the proportion of lethal recessive disorders found to be at unusually high incidences because of periodic overdominance is likely to be a small subset, the majority being more likely due to an ascertainment bias in identification (Amorim et al., 2017).

The results we have established in this work relate to the subset of Mendelian disorders corresponding to a lethal disease homozygote. Although the majority of lethal disorders are autosomal recessive conditions, such as cystic fibrosis and Tay-Sachs, it should be noted that the treatment outlined in this work can also be applied to rare dominant lethal conditions, such as achondroplasia, where individuals homozygous for the mutation are unlikely to survive infancy, unlike the non-lethal heterozygous state (Pauli et al., 1983). Thus, despite involving strong selection, such diseases are susceptible to a detailed analysis.

To summarize, we believe the results presented in this work shed new light on the possible behaviors that can occur in well-characterized genetic systems involving lethal alleles.

## Data Availability Statement

The procedures for the generation of all simulated datasets are provided in the Supplementary Material with further details deposited on GitHub (https://github.com/AndyOverall/Overdominance).

## Author Contributions

DW and AO conceived the paper. DW carried out the analyses. DW and AO co-wrote the paper.

### Conflict of Interest

The authors declare that the research was conducted in the absence of any commercial or financial relationships that could be construed as a potential conflict of interest.

## Footnotes

^1^The conventional Wright-Fisher model also assumes census and effective population sizes coincide, however it is possible to incorporate the effective population size into the Wright-Fisher model (Zhao et al., 2016).

^2^The half time to equilibrium, for a finite population, is taken as the time for the expected value of the frequency to reach the mid-point of the expected values of the frequency in the stationary distributions corresponding to the pre and post-jump values of *h*.
