# Supplementary material for: Influence of Dominance and Drift on Lethal Mutations in Human Populations
Source: Front Genet. 2020 Apr 2;11:267. doi: 10.3389/fgene.2020.00267 (PMC7142268; doi:10.3389/fgene.2020.00267)
Supplement: Supplementary file 1 [file Data_Sheet_1.PDF]

SUPPLEMENTARY MATERIAL

Influence of Dominance and Drift on Lethal Mutations in Human Populations

D. Waxman[1] and A. D. J. Overall[2]

[1]Centre for Computational Systems Biology, ISTBI,
Fudan University, 220 Handan Road, Shanghai 200433, PRC

[2]School of Pharmacy & Biomolecular Sciences, Huxley Building,
University of Brighton, Brighton, East Sussex, BN2 4GJ, UK

Correspondence to:
Professor D. Waxman
Centre for Computational Systems Biology, ISTBI,
Fudan University, 220 Handan Road, Shanghai 200433, PRC.
E-mail: davidwaxman@fudan.edu.cn

Part A: Results for a Lethal Allele in an Effectively Infinite Population

In this part of the Supplementary Material we determine a number of results including: (i) the equation obeyed by the frequency of the lethal allele in adults in an effectively infinite population, (ii) the equilibrium value of this frequency, (iii) the exact solution for the frequency as a function of time, and (iv) the 'half time to equilibrium'.

The analysis is based on a model of a diploid dioecious population with equal sex ratio. This has the following discrete-generation lifecycle, where census is made in adults.

Lifecycle

Adults<br>(start of generation)	
↓	gamete production with mutation;<br>random mating
Zygotes	
↓	death of all adults;<br>viability selection on zygotes
Juveniles	
↓	number regulation
Adults<br>(start of next generation)	

We assume each adult produces the same very large (effectively infinite) number of gametes, and that viability selection acts in zygotes at a single biallelic locus. We denote the alleles at this locus by a and A. The three possible genotypes have the relative viabilities given by Eq. (1), namely aa: 0, aA: $1-h$ and AA: 1, where a is the disease-causing allele and A the wild type allele. To maintain generality, the relative fitness of the heterozygote, $1-h$, which we simply term the *fitness of the heterozygote*, has to be non-negative ($1-h \geq 0$) but is otherwise unrestricted.

Mutations are taken to be one-way, from the A allele to the a allele, and in each generation each A allele has probability u of mutating.

To begin, we take the (relative) frequencies of the different genotypes in adults, at the start of a particular generation, termed the *present generation*, to be

Genotype	aa	aA	AA
Frequency in adults	$P=0$	Q	R

(A1)

with lethality of the aa genotype reflected by $P = 0$ and as a consequence $Q + R = 1$.

The frequency of the a allele in adults at the start of the present generation is denoted by X. We have $X = Q/2$ while the corresponding frequency of the A allele is $Y = 1 - X = 1 - Q/2$.

Dynamics of the allele frequency

In the first instance we need to formulate the dynamics of the population in terms of genotype frequencies, on which selection directly acts. However, we shall subsequently express the resulting dynamics in terms of the frequency of the a allele in adults, namely X.

Proceeding, we note that mutation occurs during gamete production, and the frequency of the a allele in gametes is

$$X^* = X + uY = X + u(1 - X) = u + (1 - u)X = u + (1 - u)\frac{Q}{2} \tag{A2}$$

while that of the A allele is

$$Y^* = Y - uY = (1 - u)(1 - X) = (1 - u)\left(1 - \frac{Q}{2}\right). \tag{A3}$$

After random mating, the frequencies of the aa, aA and AA genotypes in zygotes are

$$P^* = (X^*)^2, \quad Q^* = 2X^*Y^*, \quad R^* = (Y^*)^2, \tag{A4}$$

respectively.

The frequencies of the aa, aA and AA genotypes in juveniles, after viability selection has acted, are given by

$$P^{**} = 0 \tag{A5}$$

$$Q^{**} = \frac{(1 - h)\,Q^*}{(1 - h)\,Q^* + R^*} = \frac{2\,(1 - h)\,X^*}{Y^* + 2\,(1 - h)\,X^*} \tag{A6}$$

$$R^{**} = \frac{R^*}{(1 - h)\,Q^* + R^*} = \frac{Y^*}{Y^* + 2\,(1 - h)\,X^*} \tag{A7}$$

respectively.

When the size of the adult population is effectively infinite, the effectively infinite number of juveniles present suffer no frequency changes when non-selective thinning (or

number regulation) occurs. That is, there is no random genetic drift. Effectively, the juveniles simply proceed to become the adults of the next generation. The frequency of the aA genotype in the adults of the next generation, written Q', then obeys $Q' = Q^{**}$ while the frequency of the a allele in the next generation, written X', is

$$X' = \frac{Q'}{2} = \frac{Q^{**}}{2}. \tag{A8}$$

We can use Eqs. (A2), (A3) and (A6) to express Q^{**} in terms of the frequency of the a allele, X, of the given generation. We write this as

$$Q^{**} = 2X + 2F(X) \tag{A9}$$

where we have introduced the function

$$F(x) = \frac{(1-h)\,u - [h + (2-3h)u]\,x - (1-2h)\,(1-u)\,x^2}{[1 + (1-2h)u] + (1-2h)(1-u)x}. \tag{A10}$$

The reason for the two factors of 2 in Eq. (A9) becomes apparent when we combine Eqs. (A8) and (A9). We obtain the following equation, which determines X' in terms of X:

$$X' = X + F(X). \tag{A11}$$

The quantity $F(x)$ can then be seen to have the interpretation as the *deterministic evolutionary force* that acts on the frequency of the a allele when it has the value x.

Equilibrium allele frequency

Due to the form of $F(x)$, Eq. (A11) can be explicitly solved (see below) and generally leads to the a allele achieving a stable equilibrium frequency, which we write as $\hat{X}$. The explicit solution for $\hat{X}$, which obeys a quadratic equation, can be written as

$$\hat{X} = \frac{2\,(1-h)\,u}{h + (2-3h)\,u + \sqrt{h^2(1+u)^2 + 4\,(1-2h)\,u}}. \tag{A12}$$

Approximate equilibrium results

Following from Eq. (A12), we note that when $h = 0$ we have the exact result $\hat{X} = \frac{\sqrt{u}}{1+\sqrt{u}}$ which can be written as $\hat{X} = \sqrt{u} \times (1 + O(\sqrt{u}))$.

Additionally, it is possible to expand $\hat{X}$ of Eq. (A12) in the parameter $\varepsilon = \frac{u}{h^2}$, assuming ε is small. Small ε ($\varepsilon \ll 1$) corresponds to $|h| \gg \sqrt{u}$ and there are two cases to analyse.

Case 1: $h > 0$

When $h > 0$ we have $\frac{\hat{X}}{h(1-h)} = \varepsilon + (h^2 + h - 1)\varepsilon^2 + O\left(\varepsilon^3\right)$. This allows us to write $\hat{X} = h(1-h)\varepsilon \times (1 + O(\varepsilon))$ which can be written as $\hat{X} = \frac{(1-h)u}{h} \times \left(1 + O\left(\frac{u}{h^2}\right)\right)$.

Case 2: $h < 0$

When $h < 0$ we have $-\frac{(1-2h)\hat{X}}{h} = 1 + (1-h)\varepsilon - (h^2 - 3h + 1)(1-h)\varepsilon^2 + O(\varepsilon^3)$. This allows us to write $\hat{X} = -\frac{h}{1-2h} \times (1 + O(\varepsilon)) = -\frac{h}{1-2h} \times \left(1 + O\left(\frac{u}{h^2}\right)\right)$.

The above results appear in Eqs. (10), (11) and (12) of the main text.

Exact solution for the dynamics of the frequency

To determine the exact solution of the deterministic equation $X' = X + F(X)$ (Eq. (4) for the frequency of the a allele in an effectively infinite population, we start by writing Eq. (4) as

$$X' = X + F(X) = \frac{(1-h)u + (1-h)(1-u)X +}{1 + (1-2h)u + (1-2h)(1-u)X} \equiv \frac{a + bX}{c + dX}. \tag{A13}$$

Subtracting the equilibrium solution ($\hat{X}$) from both sides yields

$$\begin{aligned} X' - \hat{X} &= \frac{a + bX}{c + dX} - \hat{X} \\ &= \\ &= \frac{\left[a + (b-c)\hat{X} - d\hat{X}^2\right] + \left(b - d\hat{X}\right)\left(X - \hat{X}\right)}{\left(c + d\hat{X}\right) + d\left(X - \hat{X}\right)}. \end{aligned} \tag{A14}$$

The term in square brackets vanishes because $\hat{X}$ obeys $F(\hat{X}) = 0$ and this allows us to simplify Eq. (A14) to $X' - \hat{X} = \frac{\left(b - d\hat{X}\right)\left(X - \hat{X}\right)}{\left(c + d\hat{X}\right) + d\left(X - \hat{X}\right)}$ or equivalently

$$\frac{1}{X' - \hat{X}} = A + B\frac{1}{X - \hat{X}} \tag{A15}$$

where

$$A = \frac{d}{b - d\hat{X}} = \frac{(1-2h)}{(1-h) - (1-2h)\hat{X}} \tag{A16}$$

and

$$B=\frac{c+d\hat{X}}{b-d\hat{X}}=\frac{1+(1-2h)\,u+(1-2h)\,(1-u)\hat{X}}{(1-u)\left[(1-h)-(1-2h)\,\hat{X}\right]}. \tag{A17}$$

With $Z'=\frac{1}{X'-\hat{X}}$ and $Z=\frac{1}{X-\hat{X}}$, Eq. (A15) can be written as $Z'=A+BZ$. Introducing an explicit time index t so Z_t is the solution in generation t, we have $Z_{t+1}=A+BZ_t$, with solution $Z_t=\frac{A}{1-B}+B^t\left(Z_0-\frac{A}{1-B}\right)$. This corresponds to

$$X_t=\hat{X}+\left[B^t\left(\frac{1}{X_0-\hat{X}}+R\right)-R\right]^{-1} \tag{A18}$$

where we have set

$$R=-\frac{A}{1-B}. \tag{A19}$$

Equation (A18) constitutes the exact solution of $X'=X+F(X)$.

The half time to equilibrium

For the purposes of this part, we shall explicitly indicate the h dependence of $\hat{X}$, R and B by writing them as $\hat{X}(h)$, $R(h)$ and $B(h)$, respectively.

For *intermediate times*, as described in the main text, the scenario is that the dominance coefficient, h, has the value $h=0$ for a long time prior to $t=0$ (so equilibrium is achieved by $t=0$), and then there is a jump in the value of h, at time $t=0$, from $h=0$ to $h=h^*$ (with $h^*<0$). We then have $X_0=\hat{X}(0)$. If h stays at the value h^* indefinitely, then at long times X_t approaches $\hat{X}(h^*)$. The 'half time to equilibrium' is the time it takes (from time $t=0$) to reach the midpoint value $\hat{X}(0)+\left[\hat{X}(h^*)-\hat{X}(0)\right]/2=\left[\hat{X}(0)+\hat{X}(h^*)\right]/2$. We write this time as $T_{1/2}$ and its value follows from solving a special case of Eq. (A18), namely $\frac{\hat{X}(0)+\hat{X}(h^*)}{2}=\hat{X}(h^*)+\left[B^{T_{1/2}}(h)\left(\frac{1}{\hat{X}(0)-\hat{X}(h^*)}+R(h)\right)-R(h)\right]^{-1}$. The solution is

$$\left.\begin{aligned} T_{1/2} &= \frac{1}{\ln B(h)}\ln\left(\frac{R(h)-\frac{2}{\hat{X}(h^*)-\hat{X}(0)}}{R(h)-\frac{1}{\hat{X}(h^*)-\hat{X}(0)}}\right) \\ &\simeq \frac{\ln\left(\frac{|h^*|}{1+2|h^*|}\frac{1}{\sqrt{u}}\right)}{\ln(1+|h^*|)} \end{aligned}\right\}, \quad \text{intermediate times} \tag{A20}$$

where the second line is the approximation obtained by expanding the arguments of each logarithm in u, and keeping only the leading term.

For *later times*, as described in the main text, the scenario is that the dominance coefficient, h, has the value $h=h^*$ for a long time prior to a given time t_f (taken as

$t_f = 2000$ in the main text, with equilibrium achieved by time t_f). There is then a jump in the value of h, at time $t = t_f$, from $h = h^*$ to $h = 0$. We now have $X_0 = \hat{X}(h^*)$ and at long times after $t = t_f$ we have that X_t approaches $\hat{X}(0)$. With similar considerations to the case of intermediate times, we find the time it takes to reach the midpoint value (from time $t = t_0$) is

$$\left.\begin{aligned} T_{1/2} &= \frac{1}{\ln B(0)} \ln\left(\frac{R(h)+\frac{2}{\hat{X}(h^*)-\hat{X}(0)}}{R(h)+\frac{1}{\hat{X}(h^*)-\hat{X}(0)}}\right) \\ &\simeq \frac{\ln\left(1+4\sqrt{u}-8u-\frac{8u}{|h^*|}+\frac{2\sqrt{u}}{|h^*|}-\frac{2u}{h^{*2}}\right)}{2\sqrt{u}} \\ &\simeq \frac{\ln\left(1+\frac{2\sqrt{u}}{|h^*|}-\frac{2u}{h^{*2}}\right)}{2\sqrt{u}} \end{aligned}\right\}, \qquad \text{later times} \tag{A21}$$

where the second line is the approximation obtained by expanding the argument of logarithm in the numerator, in u and keeping all terms up to $O(u)$, while the denominator is the leading term in an expansion in $\sqrt{u}$; the third line discards terms that are small when $h^* = -0.01$ and $u = 10^{-5}$ or 10^{-8}, and hence applies to parameters in the vicinity of those adopted in the main text.

Part B: Results for a Lethal Allele in a Finite Population - the Modified Wright-Fisher Model

In this part of the Supplementary Material we extend the analysis presented in Part A, to determine the behaviour of the frequency of the lethal allele in a finite human population.

Notation

We shall make repeated use of binomial and multinomial random variables, and it is convenient to present here the notation we adopt and some basic properties of these random variables.

We use $\mathrm{Bin}\,(n,p)$ to denote a binomial *random variable* (*not* a distribution) with parameters n and p. The quantity $\mathrm{Bin}\,(n,p)$ equals the random number of successes on n independent trials, where p is the probability of success on each trial. Thus $\mathrm{Bin}\,(n,p)$ can take the values 0, 1, ..., n. When we encounter more than one independent binomial random variable, we shall distinguish them by a superscript, for example $\mathrm{Bin}^{(1)}\,(n,p)$ and $\mathrm{Bin}^{(2)}\,(n,p)$, to emphasize their independence.

We use $\mathbf{M}(n,\mathbf{p}) = (M_1(n,\mathbf{p}), M_2(n,\mathbf{p}), M_3(n,\mathbf{p}))$ to constitute a triplet of random numbers that make up a multinomial *random variable* (not a distribution) with parameters n and $\mathbf{p} = (p_1, p_2, p_3)$. The quantity $\mathbf{M}(n,\mathbf{p})$ contains the random number of times each of three categories are achieved on n independent trials, where $\mathbf{p}$ contains the probabilities of falling into each of the three categories on a single trial. With $i = 1$, 2 or 3, the quantity $M_i(n,\mathbf{p})$ can take the values 0, 1, ..., n, subject to $M_1(n,\mathbf{p}) + M_2(n,\mathbf{p}) + M_3(n,\mathbf{p}) = n$.

Background

In the lifecycle adopted in this work (explicitly given in Part A of the Supplementary Material), a generation starts with adults. We now take the number of adults in the population in a particular generation, termed the *present generation*, to be finite, and given by N. This number is the census population size.

The frequencies of the different genotypes in adults are as in Eq. (A1) of Part A of the Supplementary Material. That is aa: $P = 0$, aA: Q and AA: R, respectively.

Since $P = 0$ and $P+Q+R = 1$ we have $R = 1-Q$, thereby indicating that for adults there is only a single independent genotype frequency. This is a fact we will exploit in the finite population analysis.

Reproduction

Mutation occurs during gamete production and we have assumed that mutation is only from the A allele to the a allele, and in each generation each A allele has a probability of u of mutating.

Let

$$P(a|aA) = \left(\begin{array}{l} \text{the probability a randomly picked gamete carries the } a \\ \text{allele, given it was produced by an } aA \text{ genotype adult.} \end{array} \right)$$

Basic considerations lead to

$$P(a|aA) = \frac{1+u}{2}. \tag{B1}$$

Additionally, mutation of the A allele means an AA genotype adult has a non-zero probability of producing gametes that carry the a allele. In particular, the probability that a randomly picked gamete carries the a allele, given it was produced by an AA genotype adult, is

$$P(a|AA) = u. \tag{B2}$$

Related probabilities, that we have not specified, namely $P(A|aA)$ and $P(A|AA)$ are given by $1 - P(a|aA)$ and $1 - P(a|AA)$, respectively.

From the genotype frequencies given in Eq. (A1) of Part A of the Supplementary Material, the probability that a randomly picked adult has genotype aA is $P(aA) = Q$, while the corresponding probability the genotype is AA is $P(AA) = R = 1 - Q$. Thus the probability that a randomly picked gamete in a randomly picked individual carries an a allele, written $P(a)$, is

$$P(a) = P(a|aA)P(aA) + P(a|AA)P(AA)$$

$$= \frac{1+u}{2}Q + u(1-Q) = u + (1-u)\frac{Q}{2}. \tag{B3}$$

This coincides with the frequency of the a allele in an effectively infinite population (written as X^* in Part A of the Supplementary Material and given in Eq. (A2)). The corresponding probability that a randomly picked gamete carries an A allele is

$$P(A) = 1 - P(a) = (1-u)\left(1 - \frac{Q}{2}\right). \tag{B4}$$

The gametes become assembled into zygotes under random mating. We write the probabilities of a randomly picked zygote having the genotype aa, aA or AA as q_{aa}, q_{aA} or q_{AA}, respectively. Random mating entails

$$q_{aa} = [P(a)]^2, \quad q_{aA} = 2P(a)P(A) \text{ and } q_{AA} = [P(A)]^2 \tag{B5}$$

and it is convenient to collect these probabilities into the row vector $\mathbf{q}$ given by

$$\mathbf{q} = (q_{aa}, q_{aA}, q_{AA}). \tag{B6}$$

We now assume that a total of $N \times f$ zygotes are produced, where f is the mean fertility of individuals of the population. We take f to be independent of genotype and $f \geq 1$. Let the random numbers of aa, aA and AA genotype zygotes produced be given by N^*_{aa}, N^*_{aA} and N^*_{AA}, respectively. We adopt the simplest assumption, that a pair of randomly picked adults sexually produce a single offspring (zygote), with the procedure repeated Nf times, thereby producing Nf zygotes. We then have

$$(N^*_{aa}, N^*_{aA}, N^*_{AA}) = (M_1(Nf, \mathbf{q}), M_2(Nf, \mathbf{q}), M_3(Nf, \mathbf{q})) \tag{B7}$$

where $(M_1(n, \mathbf{p}), M_2(n, \mathbf{p}), M_3(n, \mathbf{p}))$ denote a triplet of numbers that make up a multinomial *random variable* with parameters n and $\mathbf{p} = (p_1, p_2, p_3)$.

Selection and number regulation

We next implement viability selection, taking the aa, aA and AA genotypes to have the viabilities V_{aa}, V_{aA} and V_{AA}, respectively, where the V's are the probabilities of surviving to reproduce, and all lie in the range $[0,1]$. Viability selection is taken to act independently on each individual zygote, and being probabilistic, viability selection contributes to the randomness in the numbers of genotypes. As an example of the randomness introduced by viability selection, consider an aA genotype individual which has probability V_{aA} of surviving viability selection and probability $1-V_{aA}$ of dying. For N_{aA} such individuals, the random number surviving viability selection is a binomial random variable with parameters N_{aA} and V_{aA}. We formalise this as follows. Let $\mathrm{Bin}^{(i)}(n,v)$, for $i=1$, 2 and 3, be three independent binomial random numbers with parameters n and v. Then after viability selection, the three genotypes are present in the population in the numbers $N_{aa}^{**}=\mathrm{Bin}^{(1)}(N_{aa}^{*},V_{aa})$, $N_{aA}^{**}=\mathrm{Bin}^{(2)}(N_{aA}^{*},V_{aA})$ and $N_{AA}^{**}=\mathrm{Bin}^{(3)}(N_{AA}^{*},V_{AA})$, respectively. Lethality of the aa genotype corresponds to $V_{aa}=0$ and hence to $N_{aa}^{**}=\mathrm{Bin}^{(1)}(N_{aa},V_{aa})=0$. Furthermore, because of the relative fitness assignments in Eq. (1) of the main text, there is a relationship between the viabilities of the aA and AA genotypes, namely $V_{aA}/V_{AA}=1-h$, but for much of the algebra we find it more transparent to use V_{aA} and V_{AA}. In terms of these viabilities, the numbers of the three genotypes present in the population, after viability selection, are given by

$$(N_{aa}^{**},N_{aA}^{**},N_{AA}^{**})=\Big(0,\mathrm{Bin}^{(2)}(N_{aA}^{*},V_{aA}),\mathrm{Bin}^{(3)}(N_{AA}^{*},V_{AA})\Big)\,. \tag{B8}$$

For many modern human populations there is a very low level of excess offspring production, beyond that needed to reproduce the number of individuals in the present generation. We shall incorporate this into the analysis by assuming that after viability selection has taken place, *no* non-selective deaths occur before the end of a generation. In such a case all post-selection zygotes proceed to become adults. Writing the numbers of the aa, aA and AA genotype adults at the start of the next generation as N'_{aa}, N'_{aA} and N'_{AA}, respectively, it follows (under the 'no non-selective deaths' assumption) that $N'_{aa}=0$, $N'_{aA}=N_{aA}^{**}$ and $N'_{AA}=N_{AA}^{**}$. Equations (B7) and (B8) then lead to

$$N'_{aA}=\mathrm{Bin}^{(2)}\left(M_2\left(fN,\mathbf{q}\right),V_{aA}\right) \tag{B9}$$

and

$$N'_{AA}=\mathrm{Bin}^{(3)}\left(M_3\left(fN,\mathbf{q}\right),V_{AA}\right). \tag{B10}$$

With $\bar{V}$ defined as

$$\bar{V} = q_{aA}V_{aA} + q_{AA}V_{AA} \tag{B11}$$

we shall make future use of three moment generating functions associated with N'_{aA} and N'_{AA}. These are

$$D(\mu, v) = E\left[e^{\mu N'_{aA} + vN'_{AA}}\right]$$

$$= \left(1 - \bar{V} + q_{aA}V_{aA}e^{\mu} + q_{AA}V_{AA}e^{v}\right)^{fN}, \tag{B12}$$

$$D_1(\mu, v) = E\left[e^{\mu N'_{aA} + vN'_{AA}} | (N'_{aA}, N'_{AA}) \neq (0, 0)\right]$$

$$= \frac{\left(1 - \bar{V} + q_{aA}V_{aA}e^{\mu} + q_{AA}V_{AA}e^{v}\right)^{fN} - \left(1 - \bar{V}\right)^{fN}}{1 - \left(1 - \bar{V}\right)^{fN}} \tag{B13}$$

and

$$D_2(\mu, v) = E\left[e^{\mu N'_{aA} + vN'_{AA}} | N'_{aA} + N'_{AA} = N\right]$$

$$= \left[\frac{q_{aA}V_{aA}}{\bar{V}}e^{\mu} + \left(1 - \frac{q_{aA}V_{aA}}{\bar{V}}\right)e^{v}\right]^{N} \tag{B14}$$

where the quantities μ and v are dummy variables, and the results for the three moment generating functions follow from standard properties of binomial and multinomial random variables (Rohatgi and Ehsanes Saleh, 2015).

Proceeding, we note that the quantity $N'_{aA} + N'_{AA}$ represents the number of adults contributed by the present generation to the next generation. From Eq. (B12) it follows that $N'_{aA} + N'_{AA}$ is a binomial random variable[1] with parameters fN and $\bar{V}$ (= the mean viability). The expected value of $N'_{aA} + N'_{AA}$ is then

$$E[N'_{aA} + N'_{AA}] = Nf\bar{V} \tag{B15}$$

and its variance is

$$\mathrm{Var}\left(N'_{aA} + N'_{AA}\right) = Nf\bar{V}\left(1 - \bar{V}\right). \tag{B16}$$

[1]To determine the distribution of $N'_{aA} + N'_{AA}$ the relevant moment generating function is $D(\mu, \mu) = E\left[e^{\mu\left(N'_{aA} + N'_{AA}\right)}\right]$. Equation (B12) yields $D(\mu, \mu) = \left(1 - \bar{V} + \bar{V}e^{\mu}\right)^{fN}$, which corresponds to the moment generating function of a binomial random variable with parameters fN and $\bar{V}$.

There are two similar ways to proceed.

Approach 1

In the first way, we note that Eq. (B15) indicates that the census population size remains, on average, constant over time if $f\bar{V} = 1$. This makes it natural to choose the mean fertility to be given by

$$f = 1/\bar{V}. \tag{B17}$$

This is equivalent to saying the absolute mean fitness of the population is unity, as befits a population of constant size. With such an approach, the number of adults in the next generation, $N'_{aA} + N'_{AA}$, will have an expected value of N, and will have fluctuations around this value of the order of $\sqrt{Nf\bar{V}\left(1-\bar{V}\right)} = \sqrt{N\left(1-\bar{V}\right)}$.

Approach 2

A second way to proceed is to calculate all statistics of N'_{aA} and N'_{AA}, when conditioned precisely on $N'_{aA} + N'_{AA} = N$. This way of proceeding leads (trivially) to $E[N'_{aA} + N'_{AA}] = N$, indicating that a condition like $f = 1/\bar{V}$ has effectively been imposed on the generation to generation dynamics. The conditioning has the additional effect of omitting any fluctuations in $N'_{aA} + N'_{AA}$.

Since the quantity of key importance to our investigation is the frequency of the a allele, or closely related, the frequency of the aA genotype, let us compare Approach 1 and Approach 2 for the mean and variance of the frequency of the aA genotype in the following generation. Explicitly, this frequency is

$$Q' = \frac{N'_{aA}}{N'_{aA} + N'_{AA}}. \tag{B18}$$

Approach 1, conditioned on $N'_{aA}+N'_{AA} \neq 0$ (conditioning is required since Q' is undefined when $N'_{aA} + N'_{AA} = 0$), leads to the exact result $E[Q'] = V_{aA}q_{aA}/\bar{V}$ which follows[2] from Eq. (B13). Approach 2, without requiring any additional conditioning (since $Q' = N'_{aA}/N$ is never undefined), directly leads to the identical result $E[Q'] = V_{aA}q_{aA}/\bar{V}$ using Eq. (B14).

We can further show with some work that Approach 1 leads to the variance[3] $\mathrm{Var}(Q') = \frac{1}{N}\frac{q_2 V_{aA}}{\bar{V}}\left(1 - \frac{q_2 V_{aA}}{\bar{V}}\right)$ with corrections of order N^{-2}, while Approach 2 leads precisely to

[2] To obtain an expression for $E[Q'] \equiv E\left[Q'|(N'_{aA}, N'_{AA}) \neq (0,0)\right]$ from Eq. (B13) we use $E\left[Q'\right] = \int_0^\infty \left[\frac{\partial}{\partial\mu}D_1(\mu, v)\right]_{\mu=-\lambda, v=-\lambda} d\lambda$. Explicit evaluation of the integral yields $E[Q'] = V_{aA}q_{aA}/\bar{V}$.

[3] To calculate the variance of Q' under Approach 1 we leave implicit that $(N'_{aA}, N'_{AA}) \neq (0,0)$. Then $\mathrm{Var}\left(Q'\right) = E\left[\left(Q'\right)^2\right] - \left(E\left[Q'\right]\right)^2$. We already know $E\left[Q'\right] = V_{aA}q_{aA}/\bar{V}$ and

$\mathrm{Var}(Q') = \frac{1}{N}\frac{q_2 V_{aA}}{\bar{V}}\left(1 - \frac{q_2 V_{aA}}{\bar{V}}\right)$. Thus Approach 1 and Approach 2 lead to variances that agree to leading order in N^{-1}, and differ only at higher order in N^{-1}.

The precise identity of the means and the closeness of the variances under the two approaches lead us to infer that the level of the fluctuations that occur in the population size, from generation to generation, under Approach 1 are not particularly important to the dynamics of the a allele, and from a practical point of view, Approach 1 and Approach 2 may be treated as being equivalent (both approaches lead to a near identical diffusion approximations, which are characterised by the mean and variance of Q'). Given this, we shall proceed with Approach 2, which is much simpler and more convenient to use than Approach 1. In particular, we note that Eq. (B14) indicates that N'_{aA} is a binomial random variable with parameters N and $p_{aA}V_{aA}/\bar{V}$, which we write as $N'_{aA} = \mathrm{Bin}\left(N, \frac{p_{aA}V_{aA}}{\bar{V}}\right)$. If follows that under Approach 2, the frequency of the aA genotype in the next generation is given as

$$Q' = \frac{N'_{aA}}{N} = \frac{\mathrm{Bin}\left(N, \frac{p_{aA}V_{aA}}{\bar{V}}\right)}{N}. \tag{B19}$$

It may be verified, using Eqs. (B3), (B4) and (B11) that in terms of the frequency of the a allele of the present generation, namely $X = Q/2$, that

$$\frac{p_{aA}V_{aA}}{\bar{V}} = 2X + 2F(X) \tag{B20}$$

where $F(x)$ is the deterministic evolutionary force that acts on the a allele's frequency in an effectively infinite population, when the frequency has the value x (see Eq. (5) of the main text). We can thus convert Eq. (B19) to an equation for the frequency of the

---

write $E\left[(Q')^2\right] = \int_0^\infty \lambda \left[\frac{\partial^2}{\partial \mu^2} D_1(\mu, v)\right]_{\mu=-\lambda, v=-\lambda} d\lambda = fNq_{aA}V_{aA}\int_0^\infty \lambda e^{-\lambda} \frac{\left(1-\bar{V}+\bar{V}e^{-\lambda}\right)^{fN-1}}{1-\left(1-\bar{V}\right)^{fN}} d\lambda + fN\left(fN-1\right)\left(q_{aA}V_{aA}\right)^2 \int_0^\infty \lambda e^{-2\lambda} \frac{\left(1-\bar{V}+\bar{V}e^{-\lambda}\right)^{fN-2}}{1-\left(1-\bar{V}\right)^{fN}} d\lambda$. Changing variables in the integrals to $z = 1 - e^{-\lambda}$ gives $E\left[(Q')^2\right] = I_1 + I_2$ where $I_1 = -fNq_{aA}V_{aA}\int_0^1 \ln(1-z) \frac{\left(1-\bar{V}z\right)^{fN-1}}{1-\left(1-\bar{V}\right)^{fN}} dz$ and $I_2 = -fN\left(fN-1\right)\left(q_{aA}V_{aA}\right)^2 \int_0^1 \ln(1-z)\left(1-z\right) \frac{\left(1-\bar{V}z\right)^{fN-2}}{1-\left(1-\bar{V}\right)^{fN}} dz$. We make free use of $f = 1/\bar{V}$ which applies under Approach 1. We have $\left(1-\bar{V}\right)^{fN} = \left(1-\bar{V}\right)^{N/\bar{V}} < e^{-N}$ and omit this negligible term in the denominators of I_1 and I_2. For I_1 we approximate $\ln(1-z)$ by $-z$, $\left(1-\bar{V}z\right)^{fN-1}$ by $\exp(-Nz)$, and extend the upper limit to ∞. For I_2 we approximate $\ln(1-z)\left(1-z\right)$ by $-z\left(1-\frac{z}{2}\right)$, $\left(1-\bar{V}z\right)^{fN-2}$ by $e^{-Nz} \times \left(1 - \frac{Nz^2\bar{V}}{2} + 2\bar{V}z\right)$, and extend the upper limit to ∞. We obtain $I_1 = \frac{1}{N}\frac{q_{aA}V_{aA}}{\bar{V}} + O(N^{-2})$ and $I_2 = \left(1-\frac{1}{N}\right)\left(\frac{q_{aA}V_{aA}}{\bar{V}}\right)^2 + O(N^{-2})$. Thus $E\left[(Q')^2\right] = \frac{1}{N}\frac{q_{aA}V_{aA}}{\bar{V}} + \left(1-\frac{1}{N}\right)\left(\frac{q_{aA}V_{aA}}{\bar{V}}\right)^2 + O(N^{-2})$ and $\mathrm{Var}\left(Q'\right) = \frac{1}{N}\frac{q_{aA}V_{aA}}{\bar{V}}\left(1 - \frac{q_{aA}V_{aA}}{\bar{V}}\right) + O(N^{-2})$.

a allele which reads

$$X' = \frac{\text{Bin}\,(N, 2X + 2F(X))}{2N} \qquad \begin{array}{l}\text{Wright-Fisher model}\\ \text{for a lethal genotype}\end{array}. \tag{B21}$$

Equation (B21) determines the dynamics of the frequency of the lethal a allele, under a stochastic process that is the direct analogue of the Wright-Fisher model. We note that two arguments of the binomial random variable in Eq. (B21) differ from what is normally encountered when selection is weak (in the sense that selection coefficients are small compared with 1). In particular, when the dynamics in an effectively infinite population is governed by $X' = X + F(X)$, then in a finite population under a Wright-Fisher model with weak selection we have

$$X' = \frac{\text{Bin}(2N, X + F(X))}{2N} \qquad \begin{array}{l}\text{Wright-Fisher model}\\ \text{for weak selection.}\end{array} \tag{B22}$$

Thus the Wright-Fisher model for a lethal genotype, Eq. (B21), differs in the placement of *two* factors of 2 in the arguments of the binomial random variable, compared with the Wright-Fisher model for weak selection, Eq. (B22).

In Figure S1 we provide a direct illustration, based on simulations, that the features of the Wright-Fisher model for lethal mutations, Eq. (B21), can yield significantly different results to a Wright-Fisher model that is based on a weak selection, Eq. (B22). We use a value of the dominance coefficient of $h = -0.2$ that corresponds to a case of overdominance where the heterozygote fitness is 20% higher than that of the non-lethal homozygote. Larger values of $|h|$ have been found applicable to sickle cell anemia (Fong et al. 2015).

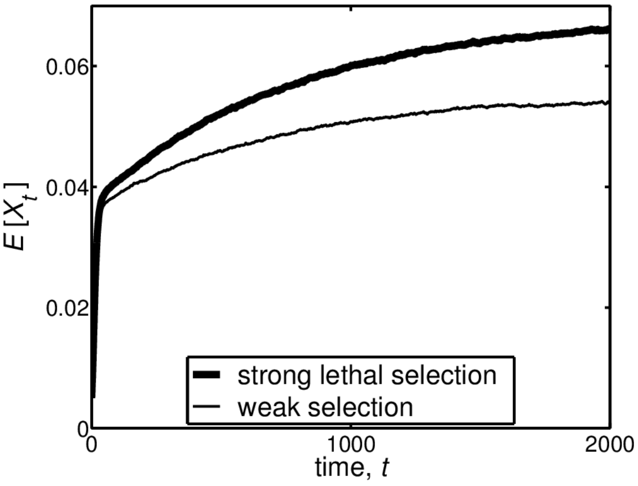

Figure S1 Caption: In this figure we compare the behaviour of the mean allele frequency, $E[X_t]$, derived from simulations of the Wright-Fisher model for a lethal mutation, Eq. (B21), with the corresponding behaviour that would be obtained from the Wright-Fisher model if a weak selection approximation applied, namely Eq. (B22). For the figure, we used a census population size of $N = 100$, an initial frequency of the lethal allele of $X_0 = 1/200$, a mutation rate of $u = 10^{-5}$, a dominance coefficient of $h = -0.2$, and 10^5 replicate trajectories.

We can gain additional insight into Eq. (B21) by showing that this equation arises from the assumptions of there being an infinite number of gametes and an infinite number of zygotes, that are implicitly made in the standard Wright-Fisher model. We proceed as in Part A of the Supplementary Material, where all equations down to Eq. (A7) are derived under the assumptions of infinite numbers of gametes and zygotes, and hence apply. We then thin the population to the census size by non-selectively picking N individuals from the population of juveniles at random. There are only two genotypes present in the population of juveniles, namely the aA and AA genotypes, and these are in the proportions Q^{**} (Eq. (A6)) and R^{**} (Eq. (A7)), respectively. Thus the number of aA genotype individuals picked is given by a binomial random number with

parameters N and Q^{**}, which in the notation used in this work is $\mathrm{Bin}(N,Q^{**})$. This yields the frequency of the aA genotype in the adults of the next generation of $Q' = \mathrm{Bin}(N,Q^{**})/N$. The frequency of the a allele in the next generation is $X' = Q'/2 = \mathrm{Bin}(N,Q^{**})/(2N)$. Lastly, by Eq. (A9), we have $Q^{**} = 2X + 2F(X)$ and we arrive at $X' = \mathrm{Bin}(N, 2X + 2F(X))/(2N)$, which is Eq. (B21).

It would thus appear that the assumption of the production of an effectively infinite number of zygotes, which is made in the standard Wright-Fisher model, is relatively innocuous in the context of lethal mutations, and makes little difference to results when the population size, N, is such that terms of order $1/N^2$ in e.g., the variance that is input into the population each generation, can be neglected compared with the leading term, which is of order $1/N$.

Part C: Diffusion analysis for a finite population

In this part of the Supplementary Material we derive the description of the frequency of the lethal allele in adults, X, in a finite population, under a diffusion approximation of Eq. (7) of the main text. Such an approximation (Kimura 1955) should be applicable for $N \gg 1$ (Kimura 1955).

With $\mathrm{Bin}(n,p)$ a binomial random variable (not a distribution) with parameters n and p, corresponding to the number of independent trials and the probability of success on a single trial, respectively, the mean is $E[\mathrm{Bin}(n,p)] = np$ and the variance is $\mathrm{Var}(\mathrm{Bin}(n,p)) = np(1-p)$. It follows that conditional on the value of X, namely that $X = x$, Eq. (7) yields

$$\begin{aligned} E[(X'-X)\,|X=x] &= E\left[\left(\frac{\mathrm{Bin}(N,2X+2F(X)}{2N} - X\right)|X=x\right] \\ &= F(x) \end{aligned} \tag{C1}$$

where $F(x)$ is given in Eq. (5) of the main text. Defining $V(x)$ as the conditional variance: $V(x) = \mathrm{Var}(X'|X=x)$ we have

$$V(x) = \mathrm{Var}\left(\frac{\mathrm{Bin}(N,2X+2F(X)}{2N}|X=x\right) = \frac{N\,[2x+2F(x)]\,[1-(2x+2F(x))]}{(2N)^2}$$

$$= \frac{[x+F(x)]\,[1-(2x+2F(x))]}{2N}. \tag{C2}$$

The diffusion approximation of Eq. (7) treats both time and the frequency of the a allele as continuous quantities, and replaces the frequency of the a allele by a continuous function of continuous time, which we write as $X(t)$. Then the function $X(t)$ incorporates randomness and obeys the stochastic differential equation

$$dX(t) = F(X(t))dt + \sqrt{V(X(t))}dW(t) \tag{C3}$$

where $W(t)$ is a Gaussian random function of time - a Wiener process (Tuckwell 1995).

Let $\phi(x,t)$ denote the probability density of $X(t)$, when evaluated at x. From Eq. (C3) it directly follows from Eq. (C3) that $\phi(x,t)$ obeys

$$-\frac{\partial\phi(x,t)}{\partial t} = -\frac{1}{2}\frac{\partial^2}{\partial x^2}\left[V(x)\phi(x,t)\right] + \frac{\partial}{\partial x}\left[F(x)\phi(x,t)\right] \tag{C4}$$

(Tuckwell 1995). This is the diffusion equation at the heart of the diffusion approximation (Kimura 1955).

Part D: Markov chain approach to the Wright-Fisher model

In this part of the Supplementary Material we present details of the Markov chain approach to Wright-Fisher model described by Eq. (7) of the main text.

We use X_t to denote the random value of the frequency of the disease-causing a allele in generation t. We also use the labels m and n to denote the number of the lethal (a) alleles in the population. Both n and m take the values $0, 1, 2, .., 2N$, and the possible frequencies of the a allele (i.e., the possible values of X_t) are given by

$$x_n = \frac{n}{2N}. \tag{D1}$$

As pointed out in the main text, the dynamics ensures that only the frequencies $x_n \leq \frac{1}{2}$ (i.e., with $n \leq N$) are actually produced in a population, and hence are possible with a lethal genotype. We thus restrict n to $n \leq N$.

We denote the probability distribution of the frequency of the a allele in generation t by the column vector $\mathbf{\Phi}(t)$. The n'th element of $\mathbf{\Phi}(t)$ is given by $\Phi_n(t) = \text{Prob}\,(X_t = x_n)$. The distribution obeys the equation

$$\mathbf{\Phi}(t+1) = \mathbf{W}\mathbf{\Phi}(t) \tag{D2}$$

where $\mathbf{W}$ is the transition matrix of the Markov chain. Elements of $\mathbf{W}$ are the conditional probabilities $W_{m,n} = \text{Prob}\,(X_{t+1} = x_m | X_t = x_n)$. While the $W_{m,n}$ are formally defined

for m and n taking the values $0, 1, 2, .., 2N$, for calculations, only the values $0, 1, ..., N$ are required. We have

$$W_{m,n} = \binom{N}{m} [2x_n + 2F(x_n)]^m [1 - 2x_n - 2F(x_n)]^{N-m} \tag{D3}$$

where $\binom{N}{m} = \frac{N!}{(N-m)!m!}$ denotes a binomial coefficient. This transition matrix differs from the form of the transition matrix of a weak selection problem with deterministic force $F(x)$, which is given by $W_{m,n} = \binom{2N}{m} [x_n + F(x_n)]^m [1 - x_n - F(x_n)]^{2N-m}$ where m and n take the values $0, 1, 2, .., 2N$.

Equation (D2) allows determination of the distribution of the a allele's frequency in different generations, thereby allowing calculation of statistics that depend on the frequency.

LITERATURE CITED IN THE SUPPLEMENTARY MATERIAL

Fong, D. C. O. C., H. Cárdenas and G. Barreto 2015 Evidence of over-dominance for sickle cell trait in a population sample from Buenaventura, Colombia. International Journal of Genetics and Molecular Biology 7: 1-7.

Rohatgi, V. K., and A. K. Md. Ehsanes Saleh 2015 An Introduction to Probability Theory and Mathematical Statistics 3rd Edition. John Wiley & Sons, New Jersey.
